# Survival and Success of Teeth Involved in Alveolar Bone Injuries: Up to 10 Years of Follow‐Up

**DOI:** 10.1111/edt.70012

**Published:** 2025-08-28

**Authors:** Cristina Braga Xavier, Anthony Marcowich Rocha, Kerian Dorothy Rehbien, Lucas Borin Moura, Letícia Kirst Post, Kauê Collares

**Affiliations:** ^1^ School of Dentistry Federal University of Pelotas Pelotas City Rio Grande do Sul Brazil; ^2^ School of Dentistry Catholic University of Pelotas Pelotas City Rio Grande do Sul Brazil

**Keywords:** alveolar process fracture, clinical study, dental trauma, survival, tooth loss

## Abstract

**Aim:**

This study aimed to describe the epidemiological characteristics of patients with ABI treated at a specialized dental trauma center between 1998 and 2023; to analyze the survival rate of teeth involved in alveolar bone injuries (ABI) and assess the risks of healing outcomes for these teeth.

**Materials and Methods:**

In total, 115 patients (367 traumatized teeth) were included in the epidemiological analysis. Among them, 47 patients (40.9%) and 145 traumatized teeth (40.8%) were followed for 12 to 118 months (1–9.8 years) after trauma. Kaplan–Meier analysis was used to evaluate the survival of traumatized teeth about pulp necrosis (PN), ankylosis‐related resorption (ARR), infection‐related resorption (IRR), pulp canal obliteration (PCO), tooth loss (TL), and the success rate of treatment. A multivariate regression analysis identified risk factors associated with these outcomes.

**Results:**

The mean follow‐up period for teeth included was 30 months. Patients with ABI comprised 11.5% of cases treated at the center. After 3 years, the TL rate was 10%; 33% of teeth exhibited no healing complications, classified as successful outcomes. PN was the most common complication, affecting 58% of the sample. The risk of TL was 15 times higher in teeth requiring repositioning or reimplantation following trauma. ABI involving alveolar bone plate avulsion was associated with increased risks of IRR, PN, and lower treatment success rates.

**Conclusions:**

Teeth involved in ABI generally have a favorable prognosis, with 90% retained in the oral cavity after 3 years. One‐third of these teeth showed no complications during the follow‐up period. However, teeth associated with alveolar bone avulsion or requiring replantation or repositioning had higher risks of complications, including IRR, PN, and TL.

## 1 Introduction

Alveolar bone injuries (ABI) can involve several teeth, and it is a complex type of traumatic injury that can affect pulp tissues, periodontal ligament, bone, and gingival tissues. It, compared to other types of TAD, has received limited attention in the literature. There are few clinical studies about this trauma, with limited data on the epidemiological aspects of ABI as well as on the prognosis of the affected teeth [1, 2, 3, 4, 5, 6].

There was a low related prevalence of this kind of trauma, ranging from 2% to 4% of cases in reports found in the literature [2, 6]. Pulp necrosis (PN) is the most frequently reported outcome in teeth involved in ABI, and certain risk factors appear to increase the likelihood of this healing complication. These factors include teeth with fully developed roots, incomplete repositioning of the dental element, fracture lines directly related to the apex [5], and concomitant injuries [6].

Survival rates for other outcomes, such as pulp calcification, resorption, and tooth loss, are relatively high, suggesting a favorable prognosis for these teeth when appropriately managed [1, 2, 6]. However, no studies assessed risk factors associated with these outcomes; highlighting the need for further research in this area.

To date, few studies have specifically focused on ABI, and even fewer have evaluated long‐term outcomes using appropriate statistical methods that account for patient‐level clustering. Additionally, injuries involving avulsion or loss of one of the bone plates are often grouped with other trauma types, despite their distinct severity. Then, this study aimed to analyze the survival rate of teeth involved in ABI and assessed the risks of healing complications with these outcomes: PN ankylosis‐related resorption (ARR), infection‐related resorption (IRR), pulp canal obliteration (CP), and tooth loss (TL) and evaluated the success rate of treatments for these teeth. Additionally, this study aimed to describe the epidemiological characteristics of patients who suffered ABI, treated for 24 years at a Reference Service for TAD Treatment. Our hypothesis was that the time interval between trauma and treatment, the type of bone injury, and the degree of displacement of the affected teeth may increase the risk of complications in these teeth, and that the prevalence of ABI is notably high in the studied healthcare service.

## 2 Methodology

The reporting of this study followed the Reporting of studies Conducted using Observational Routinely‐collected Data (RECORD) guidelines [7].

### 2.1 Study Design and Setting

This study was designed as a retrospective cohort study and represents a subset of a larger project aimed at assessing outcomes and characteristics of dental trauma cases. In the present study, patient selection was based on a detailed review of clinical records, with trauma classification following the criteria established by Andreasen and Andreasen (1995), and the IADT guidelines relevant to each time period; all patients who suffered bone alveolar injuries were included. The study was conducted using secondary data from patient records archived at the Center for the Study, Treatment, and Monitoring of Trauma in Permanent Teeth (CETAT) at the Federal University of Pelotas. Patient records from January 1998 to June 2023 were revised.

CETAT is a service involving undergraduate and graduate students as well as professors from different clinical dental specialties. All patients at the center are managed by a multidisciplinary team, including specialists in oral surgery, endodontics, operative dentistry, implantology, and prosthodontics, as well as nurses and a social worker. It has become a reference center in the southern macroregion of Rio Grande do Sul, Brazil, for the treatment of patients with trauma to permanent teeth, currently serving a population of approximately 1,000,000 people (https://cosems.com/rs/analisesaude/app/macro).

### 2.2 Data Source

Patient records from the CETAT service were used as the data source for this study. These records were completed at the time of patient care. During the initial clinical examination, the following information was collected for each patient: gender, age, cause of injury, date of trauma, affected teeth, involvement of the alveolar bone, traumatized tooth, and type of dental trauma, documented according to the TAD classification by Andreasen et al. [8]. Clinical examinations were conducted by operators under the supervision of service coordinators, including a thorough evaluation of the face and each tooth involved in the trauma, following standardized protocols for assessment, treatment, and clinical follow‐up based on the International Association of Dental Traumatology (IADT) guidelines relevant to each period [9, 10, 11, 12, 13, 14, 15, 16, 17].

All data were recorded in patient files at the end of each visit and reviewed by supervising clinicians on the same day. Periapical radiographs of the traumatized teeth were taken at all initial and follow‐up visits. Periapical radiographic examinations were performed using 70 kV, 7 to 10 mA, 1 mm total aluminum equivalent filtration, and 0.4–0.6 s exposure time (Timex 70C; Gnatus—Ribeirão Preto, SP, Brazil). E‐speed films (Kodak) 3 × 4 cm in size were used. All radiographs were processed by the time–temperature method in a dark room, and standardized positioning devices were used, ensuring at least one orthoradial view of the traumatized tooth. When necessary, additional images were taken with varying horizontal and/or vertical angulation. Before being filed, radiographs were scanned and digitally stored. Beginning in 2006, photographic records of the oral cavity with frontal and profile views were also taken for all patients with trauma, using a digital camera, and from 2018 onward, a smartphone camera. These images were subsequently archived in individual digital folders for each CETAT patient.

The identification of exposures, trauma types, and outcomes did not rely on electronic codes or automated algorithms. Instead, all variables were recorded manually by trained clinicians based on clinical and radiographic criteria, and classified according to established dental trauma.

### 2.3 Participants and Inclusion Criteria

**FIGURE 1:**
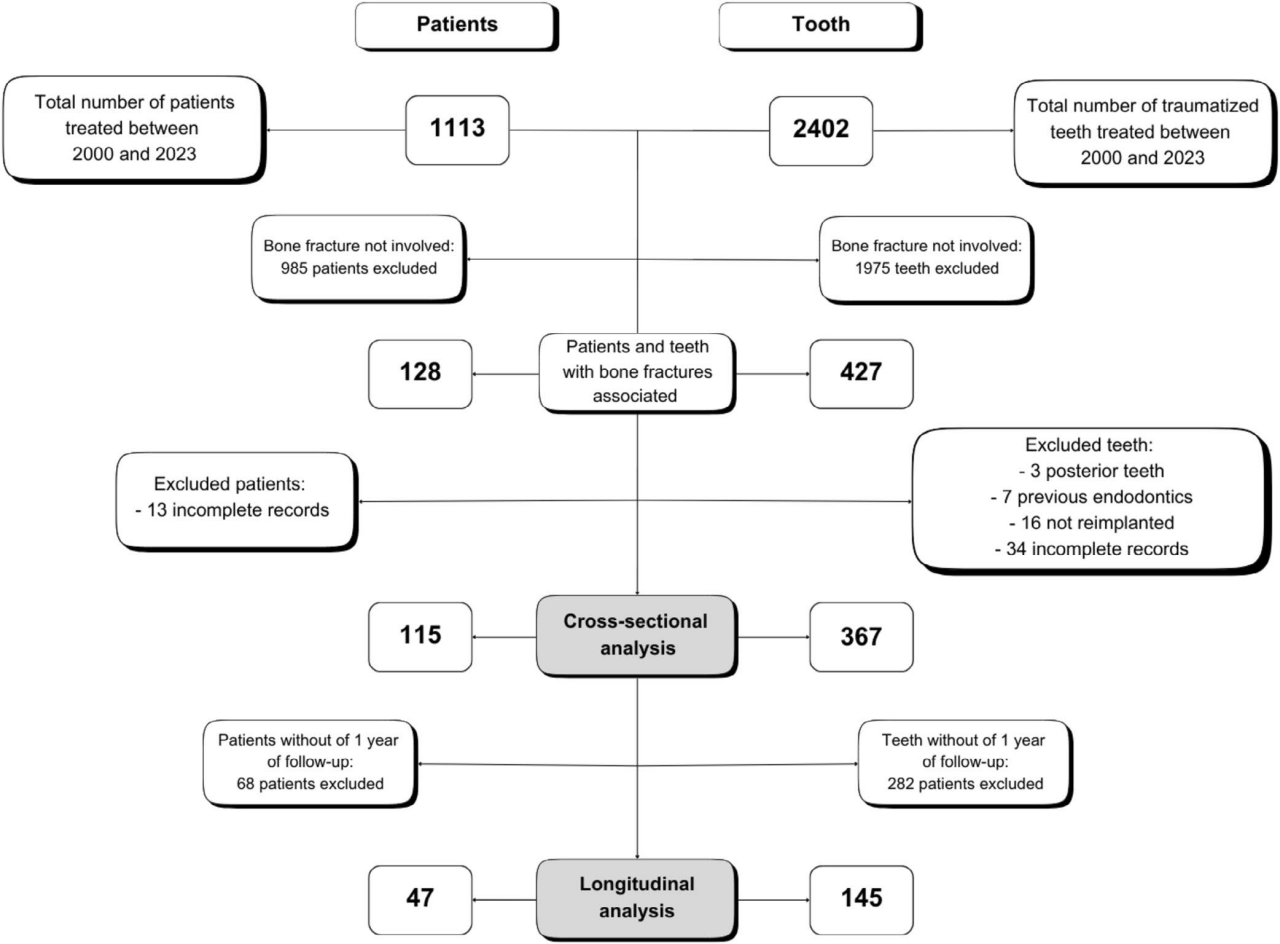
Flowchart describing patients treated at CETAT and the process of inclusion and exclusion of these in the study.

The study was structured in two phases (Figure 1):

1. First Phase—Cross‐Sectional Analysis: This phase involved a cross‐sectional analysis of patients with ABI, aiming to determine the epidemiological profile and characterize the initial trauma treatment. The inclusion criteria for this phase involved records in which the field “associated ABI” was duly completed, indicating the occurrence of trauma. Exclusion criteria, at the patient level, included records that did not specify the teeth involved in the fracture, did not contain the patient's initial radiograph, cases where the trauma occurred in deciduous teeth, and records of patients with mandibular and/or maxillary fractures requiring hospital intervention for rigid internal fixation. At the tooth level, exclusions included teeth that were not reimplanted, posterior teeth, and those that had undergone endodontic treatment before the trauma.
2. Second Phase—Longitudinal Analysis: In the second phase, a longitudinal analysis was conducted to evaluate the primary outcomes of bone trauma in patients treated and monitored in the service, as well as to identify risk factors associated with these outcomes. Inclusion criteria for this phase involved patients with a minimum follow‐up period of 1 year in the service, records containing complete clinical and radiographic documentation from the initial consultation and periodic follow‐ups, and patients with permanent teeth in the fractured segment. Exclusion criteria included patients who only attended the initial consultation in the service, those with a follow‐up period shorter than 1 year, and patients with a history of previous trauma in the same region. Additionally, teeth that experienced new trauma in the region before completing the first year of follow‐up, those with prior endodontic treatment, and avulsed teeth that were not reimplanted (lost) were also excluded.

### 2.4 Variables

For the cross‐sectional analysis, the following variables were considered: gender (male and female), patient age (collected in years and categorized into three groups: 6–12 years, 13–18 years, and over 18 years), trauma etiology (categorized into four main groups: falls, violence, traffic accidents, and others, as mentioned by Andreasen and Lauridsen [2]); date of trauma and date of first attendance (collected in hours and categorized into groups: on the day of the trauma, within 24 h post‐trauma, from 1 to 7 days post‐trauma, and more than 7 days), trauma region (anterior or posterior), involved dental arch (upper and lower), number of teeth involved in the trauma (categorized into terciles: 1–3 teeth, 4–6 teeth, and 7–9 teeth), number of teeth directly involved in the fracture line (categorized into terciles: 1–2 teeth, 3–4 teeth, and 5–6 teeth), type of bone fracture (classified based on the modification of the classification by Andreasen et al. [18]), which considers three groups: fracture of one of the alveolar walls (when the fracture line is confined to a single alveolar wall, either vestibular or palatal/lingual), avulsion of one of the alveolar walls (when one of the bony walls is detached and cannot be repositioned), and fracture of the alveolar process (when the fracture line involves the entire alveolar process, including septal fractures; comminution/smashing was not considered due to the difficulty of imaging diagnosis), fracture location (involving the periodontal ligament or not), dental group involved (central incisors, lateral incisors, and canines), and type of associated dental trauma (collected according to the classification by Petti et al. [19]), categorized into three groups: trauma to dental tissues (coronal, corono‐radicular, and radicular fractures), trauma to supporting tissues (concussion, subluxation, extrusive, intrusive, and lateral luxations), and avulsions (avulsed and replanted teeth).

For the longitudinal analysis, all the aforementioned variables were considered, along with the use of orthodontic appliances at the time of trauma (yes or no), presence of concomitant root fracture at the fracture line (yes or no), degree of root development (complete and incomplete), use of retention (yes or no), retention period (collected in days and categorized into two groups: up to 60 days or more than 60 days), and whether re‐implantation or dental repositioning was performed (yes or no).

### 2.5 Outcomes

The outcomes considered for the study were evaluated during follow‐up visits, following the standard protocol for trauma follow‐up times recommended by the IADT. Dates and data from all patient consultations in the project were collected. The final evaluation period corresponded to the last visit when radiographs of the affected teeth were performed; generally, this date coincided with the patient's final visit to the service. All evaluated outcomes are described below:

1. PN: PN was considered in teeth that underwent endodontic treatment after trauma. To define PN in the service, at least two clinical parameters had to be present, such as signs of acute infection or fistula, absence of response to pulp sensitivity tests, discoloration of the crown, presence of external inflammatory resorption, or periapical lesion. In cases of doubt, cavity tests were performed without anesthesia; if painful sensitivity or abundant bleeding was confirmed and the pulp showed adequate consistency, the procedure was interrupted, dressings were applied, and the tooth continued to be monitored. Additionally, replanted teeth with complete root development were indicated for endodontic treatment 7–14 days after replantation.
2. External Inflammatory Resorption: Cases were considered where irregular radiolucent areas appeared along the cementum and dentin, typically of rapid progression and often inhibited or halted by treatment with calcium hydroxide.
3. Substitutive Resorption: Teeth were considered when radiographic analysis revealed the disappearance of the lamina dura space and/or part of the root surface was replaced by bone tissue. Records of total absence of mobility and a metallic sound upon percussion were observed, with or without infra occlusion during clinical examination.
4. Root Canal Calcification: Teeth were considered where radiographic analysis showed that part or all of the root canal and pulp chamber lumen were replaced by a dentin‐like structure compared to the initial radiograph, associated or not with changes in crown coloration during clinical examination.
5. Tooth Loss: Tooth loss was counted on the day the extraction was performed or at the subsequent consultation when the patient arrived without the tooth. Patients who arrived without the tooth or whose tooth needed to be removed at the first visit to the Center were excluded from the study.
6. Success: Success in this study was defined as the absence of any of the aforementioned adverse outcomes at the time of evaluation. The tooth was present and functional, exhibited pulp vitality, and showed no signs of root canal calcification or resorption, indicating periodontal healing.

### 2.6 Data Analysis

All data were collected independently by two trained researchers (KR and AMR) using Microsoft Excel spreadsheets at different time points. The datasets were then compared, and any discrepancies were reviewed and resolved by the senior researcher (CBX). Statistical analyses was performed using the STATA 14 software package (StataCorp LP; College Station, TX, USA). Descriptive statistics were used to report the frequency distribution of patients and teeth involved in bone injuries by independent variables. The longevity of these teeth was explored by Kaplan– Meier statistics and survival tables. Annual failure rates (AFRs) were calculated from survival tables according to the formula: (1 − *y*) *z* = (1 − *x*), in which “*y*” expresses the mean AFR and “*x*” the total events rate at “*z*” years. The proportional hazards test was assessed for each variable. For each outcome, we verified the number of observed events to ensure the stability of the multivariable Cox regression models. To reduce the risk of overfitting, only variables that demonstrated a *p*‐value < 0.20 in the univariate analysis were considered for inclusion in the final models, in line with recommendations for exploratory studies with limited sample sizes. When the number of events for a specific outcome was insufficient to meet minimum modeling assumptions, only univariate Cox regression was performed. Cox proportional hazards models with shared frailty (gamma distribution) were used to account for clustering of teeth within patients.

### 2.7 Data Access and Quality Control

The clinical data was independently extracted and entered by two calibrated researchers (KR and AMR) using standardized spreadsheets. Discrepancies were reviewed and resolved by a senior researcher (CBX). Only these three individuals had access to the complete dataset. Data were verified for internal consistency, and missing or ambiguous entries were documented without imputation. No linkage with external databases was performed.

## 3 Results

Over the 24 years of follow‐up, a total of 1113 patients were treated, encompassing 2402 traumatized permanent teeth. Among these patients, 128 experienced some form of alveolar bone injury, representing 11.5% of the treated population and involving 427 permanent teeth (17.8%) in the trauma. After applying inclusion and exclusion criteria, a total of 115 patients and 367 traumatized teeth (10.3% of patients and 15.3% of traumatized teeth) were included in the first stage of the study. In the second stage, 47 patients (40.9%) and 145 traumatized teeth (40.8%) remained in follow‐up at the center, with a follow‐up period ranging from 12 months (1 year) to 118 months (9.8 years), resulting in an average follow‐up time of 30 months (2.5 years). Figure 1 provides further details on the included data.

Most patients were male (89 patients—77.4%), with ages ranging from 6 to 63 years; patients over 18 years of age represented 64% of the cases. The main cause of trauma was motor vehicle accidents (38.2%), followed by interpersonal violence (27%). The most frequent type of bone trauma was alveolar process fracture (47.3%), and it was observed that 47% of patients had 1–2 teeth directly involved in the bone injury area. Central incisors were the most affected teeth, representing almost half of the dental elements involved in the trauma (48.2%), and injuries affecting the supporting tissues were the most prevalent (150 teeth—40.9%). Variable proportions remained similar between the two analyses, with no significant modifications in the sample profile. The complete description of the data collected is available in Table 1.

**TABLE 1:** Descriptive analysis of demographic data and tooth/trauma characteristics for patients and teeth in alveolar bone injuries (cross‐sectional‐367 teeth in 115 patients; Longitudinal—145 teeth in 47 patients).

	Cross‐sectional	Longitudinal
*Patient‐related variables*	115 patients (100%)	47 patients (40.9%)
Gender		
Female	26 (22.6)	11 (23.4)
Male	89 (77.4)	36 (76.6)
Patient age*		
7–12	18 (15.8)	10 (21.7)
13–18	23 (20.2)	11 (23.9)
18 or over	73 (64.0)	25 (54.4)
Trauma etiology		
Falls	21 (18.3)	10 (21.3)
Traffic accidents	44 (38.2)	17 (36.1)
Violence	31 (27.0)	10 (21.3)
Others	19 (16.5)	10 (21.3)
Number of teeth involved in the trauma (tertile)		
1–3	59 (51.3)	18 (38.3)
4–5	39 (33.9)	23 (48.9)
6–9	17 (14.8)	6 (12.8)
Arch involved in trauma		
Only 1	—	33 (70.2)
Both	—	14 (29.8)
First attendance		
On the day	10 (8.8)	5 (10.6)
1–7 days after trauma	53 (46.5)	24 (51.1)
After 1 week	51 (44.7)	18 (38.3)
Orthodontics in treatment		
No	—	40 (85.1)
Yes	—	7 (14.9)
Type of bone fracture**		
Board fracture	49 (43.8)	16 (37.2)
Block fracture	53 (47.3)	20 (46.5)
Avulsion	10 (8.9)	7 (16.3)
Number of teeth involved in the fracture (tertile)		
1–2	54 (47.0)	16 (34.0)
3–4	50 (43.5)	27 (57.4)
5–7	11 (9.5)	4 (8.6)
Early tooth loss		
No	106 (92.2)	38 (80.8)
Yes	9 (7.8)	9 (19.2)
*Tooth‐related variables*	367 teeth (100%)	145 teeth (39.5%)
Tooth type		
Central	177 (48.2)	69 (48.6)
Lateral	138 (37.6)	58 (40.0)
Canine	52 (14.2)	18 (12.4)
Dental arch		
Upper	207 (56.4)	80 (55.1)
Lower	160 (43.6)	65 (44.8)
Dental trauma type		
Dental	145 (39.5)	54 (37.2)
Support	150 (40.9)	73 (50.3)
Avulsion	72 (19.6)	18 (12.4)
Root fracture		
No	309 (84.2)	125 (86.2)
Yes	58 (15.8)	20 (13.8)
Periodontal ligament involvement***		
No	203 (55.6)	47 (32.9)
Yes	162 (44.4)	96 (67.1)
Rhizogenesis		
Complete	+	139 (95.9)
Incomplete	+	6 (4.1)
Use of dental retainer****		
No	+	42 (30.0)
Yes, up to 60 days	+	35 (25.0)
Yes, more than 60 days	+	63 (45.0)
Reimplantation/repositioning		
No	+	118 (81.4)
Yes	+	27 (18.6)

*Note:* —: Missing data; *: one patient; **: four patients; ***: two teeth; ****: five teeth; +: variables not collected in the first service.

Table 2 provides the survival and annual failure rates for the six distinct outcomes evaluated in this study. At the 3‐year follow‐up, it was observed that 9 out of 10 teeth remained in the oral cavity. A success rate of 64% was observed at 6 months of follow‐up, and after 3 years, 33% of the traumatized teeth were still completely intact. Among the outcomes studied over the 3‐year follow‐up, PN was the most frequent, accounting for 58% of cases. IRR was observed in 20% of cases; ARR in 10%; and PCO in 2% of cases within the first 6 months, increasing to 22% at 3 years.

**TABLE 2:** Survival rate and annual failure rate (AFR) of success and healing complications for teeth in alveolar bone injuries (47 patients and 145 traumatized teeth).

Outcomes	6 months		12 months		24 months		36 months	
	Estimat.	AFR	Estimat.	AFR	Estimat.	AFR	Estimat.	AFR
Pulp canal obliteration	98%	3.96	95%	5.00	91%	4.61	78%	7.95
Ankylosis‐related resorption	97%	5.91	93%	7.00	92%	4.08	90%	3.45
Tooth loss	94%	11.64	92%	8.00	90%	5.13	90%	3.45
Infection‐related resorption	90%	19.00	85%	15.00	83%	8.90	80%	7.17
Pulp necrosis	73%	46.71	71%	29.00	70%	16.33	58%	16.60
Success^a^	64%	59.04	56%	44.00	49%	30.00	33%	30.90

^a^ Tooth that did not show any complications (outcome) in period.

**FIGURE 2:**
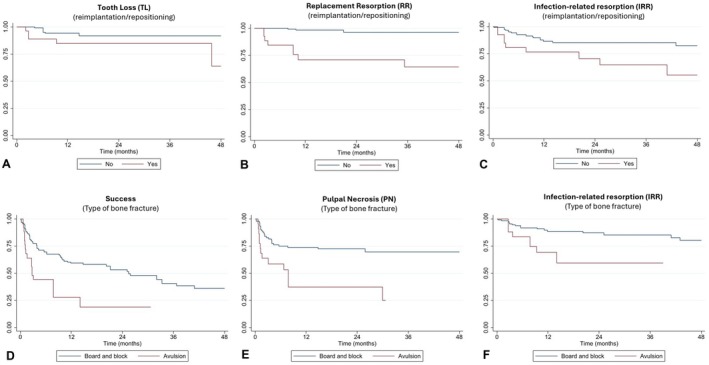
Kaplan–Meier survival curves. (A) Tooth survival (TL) in teeth that were reimplanted/repositioned versus those that were not. (B) Replacement resorption (RR) survival in reimplanted/repositioned teeth versus non‐reimplanted/non‐repositioned teeth. (C) Infection‐related resorption (IRR) survival in reimplanted/repositioned teeth versus non‐reimplanted/non‐repositioned teeth. (D) Infection‐related resorption (IRR) survival according to the type of alveolar bone fracture. (E) Pulp necrosis (PN) survival according to the type of alveolar bone fracture. (F) Survival of favorable outcomes according to the type of alveolar bone fracture.

Table 3 presents the results of the multivariable regression models for the outcomes of success and healing complications. The analyses showed that the risk of tooth loss was 15 times higher in teeth that were repositioned or reimplanted after trauma compared to those without these interventions (Figure 2A). Exposure to this intervention also increased the risk of ankylosis by 23 times (Figure 2B) and inflammatory resorption by 2.6 times (Figure 2C). Alveolar bone avulsion (HR 3.4) was also associated with a higher risk of RI (HR 3.3) (Figure 2F) and PN (HR 3.7) (Figure 2E), reducing the chances of success (HR 3.4) (Figure 2D). The estimated variance of the frailty term (θ) ranged from 0.40 to 23.5 across the models, indicating varying degrees of heterogeneity between patients. Other variables evaluated showed an increased risk for some of the outcomes and are detailed in Table 3.

**TABLE 3:** Results of multivariate Cox's regression models with shared frailty for variables related to success and healing complications (*N* = 145 teeth in 47 patients).

	Success^a^		Pulp canal obliteration^b^		Ankylosis‐related resorption^b^		Infection‐related resorption		Pulp necrosis^c^		Tooth loss^d^	
	HR (95% CI)	*p*	HR (95% CI)	*p*	HR (95% CI)	*p*	HR (95% CI)	*p*	HR (95% CI)	*p*	HR (95% CI)	*p*
*Patient‐related variables*												
Age (ref = 7–12)										0.017		
13–18									10.7 (1.5–68.9)			
18 or over									5.2 (0.8–32.2)			
Type of bone fracture (ref = Board and block)		0.002						0.020		0.039		
Avulsion	3.4 (1.5–7.4)						3.3 (1.2–9.1)		3.7 (1.1–12.7)			
*Tooth‐related variables*												
Tooth (ref = Lateral/canine)		0.002				0.023				0.006		
Central	2.2 (1.3–3.6)				6.3 (1.3–31.3)				2.8 (1.3–5.8)			
Dental arch (ref = lower)						0.031						
Upper					10.6 (1.2–90.4)							
Dental trauma type (ref = Dental)				0.033		< 0.001						
Support			30.5 (2.3–402.1)		1.43 (0.1–17.4)							
Avulsion			4.1 (0.2–100.6)		52.9 (5.1–545.4)							
Reimplantation/Repositioning (ref = no)						< 0.001		0.040				0.020
Yes					23.0 (4.6–114.8)		2.6 (1.0–6.6)				15.2 (1.5–150.1)	
Theta (variance of frailty term)	3.5		9.3		3.4		0.4		6.3		23.5	

Abbreviations: CI, confidence interval; HR, hazard ratio; ref., reference.

^a^ Tooth that did not show any outcome in the period.

^b^ Result of univariate analysis. The outcome is not sufficiently flawed to perform multivariate analysis.

^c^ For the pulp necrosis outcome, 114 teeth were evaluated, considering that 31 teeth underwent endodontics at the beginning of follow‐up.

^d^ Result of univariate analysis. Only one variable is associated with the outcome.

## 4 Discussion

The findings of this study indicate that, despite the severe initial impact of trauma involving multiple tissues, the prognosis for teeth preserved immediately after injury is favorable. After 3 years of follow‐up, 90% of traumatized teeth remained present. Moreover, 64% of teeth exhibited no adverse outcomes, classified as successful within 6 months. By 3 years, one‐third of these teeth were completely healthy. Previous studies have similarly reported low rates of healing complications in teeth affected by such trauma [6, 9, 10], although their methodologies differ somewhat from the present study. A key strength of this study lies in its exclusive focus on ABI cases, with a follow‐up period of up to 10 years. The separate analysis of severe injury types—such as avulsion and loss of a bone plate—demonstrated their association with increased risk of inflammatory resorption, PN, and reduced clinical success. The statistical model identified tooth repositioning/reimplantation as the only variable significantly associated with an increased risk of tooth loss (TL). This finding likely reflects the severity of the trauma, as these procedures are typically required for teeth with severe displacement and associated periodontal and pulpal injuries, which worsen the prognosis over time [5, 11, 12, 13, 14, 15, 16, 17, 20, 21].

TL estimated annual failure was 8% in the first year, decreasing to 3.45% by the third year. This suggests that teeth with poor prognosis are often lost early while surviving teeth tend to stabilize in the oral cavity. Previous studies have reported an estimated risk of tooth loss of 7.8% over 10 years of follow‐up for teeth with complete root development [5] and 4% over 5 years [6]. In this study, teeth lost during the initial trauma or extracted at the first appointment due to non‐viability were excluded from the survival analysis. Patients were also excluded unless they had additional traumatized teeth that were retained (*n* = 9 teeth). This approach aligns with Lauridsen and Marotti but differs from Freihofer's 1969 study, which reported higher TL rates by including early extractions as losses.

PN was the most frequent healing‐related outcome, consistent with findings by Andreasen [1], Lauridsen et al. [5], and Marotti et al. [6]. Over 50% of teeth, however, remained vital after 3 years. The risk of PN was highest within 6 months post‐trauma (46 times), declining to 16.6 times after 3 years. Lauridsen [5] similarly reported a 56% risk of PN in teeth with complete root development over 10 years. PN definitions vary, incorporating clinical and radiographic signs such as lack of pulp response [2, 4], periapical lesions [2, 3, 4, 5, 6], fistulas, abscesses, discoloration, and pain [5]. Most studies required at least two signs for diagnosis, with cavity testing in uncertain cases [1, 6]. In this study, replanted teeth with complete root formation were classified as non‐vital due to endodontic treatment performed 7–10 days post‐trauma per IADT guidelines [11, 12, 13, 14, 15, 16, 17].

Regarding other healing outcomes, a decline in the survival rate of teeth with intracanal calcification was observed between 24 and 36 months, dropping from 91% to 78%. This aligns with Lin et al. [22], who reported that calcifications are more frequently detected in teeth with longer follow‐up periods. Marotti et al. [6] observed higher rates of root canal calcification over time, interpreting it as a pulpal response to injury. However, this was not considered a severe complication, as it does not lead to tooth loss but rather represents a silent sequela, often only identified when it causes discoloration [23]. Andreasen [1] noted that intracanal calcification rates may be underreported due to the high incidence of PN and subsequent endodontic treatment, which would exclude this outcome.

In this study, avulsed and luxated teeth showed a significantly increased risk of PC and RR compared to those with isolated dental fractures. However, teeth with minimal trauma to supporting tissues, such as concussion or subluxation, were grouped with luxations. This grouping may have underestimated outcomes in the follow‐up and contributed to the increased risk of pulp calcifications in this category [24]. Teeth with concomitant dental and supporting tissue trauma were categorized under the most severe trauma type [15, 16, 17]. While this grouping may introduce bias, it was necessary due to sample size limitations to allow for risk estimation. These findings should therefore be interpreted with caution.

To minimize bias, this analysis focused on the type of treatment performed immediately after trauma (repositioning/reimplantation) as an indicator of trauma type and severity, as only teeth with avulsions or severe luxations (lateral, intrusive, or extrusive) required these interventions. Data were obtained from the service and a referral hospital where most patients received initial care. Repositioning or reimplantation was associated with increased risks of TL, IR, and RR. These risks stem from extensive tooth displacement, causing significant damage to the periodontal ligament (PDL) and cementum. Damage exceeding 20% of the root surface predisposes to cellular necrosis and RR [25, 26]. RR can also result from alveolar bone crushing during lateral or intrusive luxations, where clastic cells replace dentin with bone [21]. IR is primarily linked to bacterial contamination and necrotic pulp. At 3 years, survival rates were 90% for RR and 80% for IR, with annual failure rates decreasing over time. These findings align with Andreasen and Lauridsen [2, 5], who reported low resorption rates.

Another important finding of this study was the association between alveolar bone avulsion and poorer outcomes. Although not the most frequent type of fracture, it occurred in 16.3% of cases and was significantly associated with increased risks of PN, RI, and reduced treatment success. This may be attributed to the severity of the trauma, leading to bone detachment and reduced protection of dental tissues. These findings align with the bone defect healing model (1‐, 2‐, or 3‐wall defects), which demonstrated that better preservation of bone walls improves periodontal healing and regeneration [27]. Bone fractures were classified using a modified Andreasen system [18]. This approach confirmed that bone avulsion had significantly the worst outcomes. At the same time, other fracture types showed no increased risk for complications or evidence of infection or bone sequestration, consistent with favorable healing when properly managed [5]. This study excluded mandibular and maxillary fractures requiring surgical access and fixation, as these cases often involve additional treatments, such as intermaxillary fixation, and delayed dental management, which could introduce bias into the analysis.

Several variables were analyzed in this study using a univariate regression model to explore their association with the outcomes. We recognize that some healing outcomes had a limited number of events, which may reduce the precision of estimates and increase the risk of overfitting. To mitigate this, we limited the number of covariates per model; for rare outcomes, only univariate Cox regression was performed. Most findings aligned with trends in the literature. Teeth in patients older than 13 showed a higher risk of PN, likely due to incomplete apical closure by this age and the guidelines recommendation for endodontic treatment of avulsed teeth 7–10 days post‐reimplantation due to minimal chances of pulp revascularization [14, 16]. Central incisors (CI) were the most frequently affected teeth and showed higher chances of success compared to lateral incisors (LI) and canines (C), as they avoided unfavorable outcomes over the study period. However, they also had a higher risk of RR and PN, possibly due to their increased exposure to trauma [28, 29]. Most ABI occurred in the maxilla, with 29.8% of patients having involvement in both arches; however, this variable and others related to immediate treatment, such as splint use, splint duration, and orthodontic appliance use during trauma, showed no association with the studied outcomes.

Methodological challenges can arise when comparing results and evaluating outcomes, particularly in cases of combined dental trauma [19, 22]. Lauridsen et al. [30] reported an increased risk of PN in teeth with simultaneous injuries; however, ABI or combined traumas involving multiple teeth in the same patient were not considered. In this study, the number of teeth involved in alveolar fractures and those directly associated with the fracture line were evaluated. Neither analysis showed a significant association with healing outcomes. Most patients treated at the center had 1–2 teeth affected (47%), but those who continued follow‐up often had 3–4 teeth involved (57%), suggesting better adherence in cases of more severe trauma. This contrasts with findings from another center, where the average number of traumatized teeth was three, but follow‐up cases mainly involved patients with up to two affected teeth [6].

This study presents several limitations inherent to retrospective designs. Most patients initially received care at a maxillofacial trauma referral hospital before being referred to our center. As only 10% were seen on the day of trauma, important clinical variables—such as soft tissue injuries, antibiotic use, and time to repositioning—could not be fully analyzed due to incomplete records. Additionally, recommended IADT follow‐up intervals were not always followed, given service demands and individualized care needs. These factors may contribute to selection bias, as patients with more severe trauma were more likely to continue long‐term follow‐up, potentially overestimating complication rates. Misclassification bias is also possible due to variability in trauma classification and outcome registration over the 24‐year period, despite the use of standardized protocols. Furthermore, follow‐up bias may have occurred, as patients with milder injuries often discontinued care earlier, possibly leading to underestimation of late complications. Diagnostic limitations also apply, as conventional radiography was used instead of CBCT, which may have reduced sensitivity for detecting minor fractures. Future studies using advanced imaging and standardized follow‐up protocols may help overcome these limitations.

A notable strength of this study was the high percentage of alveolar fractures, accounting for 11.5% of all patients treated over 24 years. This rate exceeds those of other centers, where alveolar fractures represent 3.4% and 2% of cases [2, 6]. However, only 40.9% of cases were followed for the minimum period outlined; although the epidemiological profile remained consistent in both analyses, suggesting the sample's representativeness. The high prevalence of this trauma type may be linked to local characteristics, enabling a robust analysis of the various factors involved. This helped identify risk factors for healing outcomes and success rates of affected teeth. We chose to present data on all patients with ABI, alongside those followed at the center, as even recent studies lack detailed information on alveolar fractures and associated risks.

## 5 Conclusion

Patients with ABI represented 11.5% of cases treated at the service, with the majority being men over 18 years old, primarily affected by motor vehicle accidents. Most cases involved one or two teeth directly associated with the bone injury area, and alveolar process fractures were the most frequently observed type of bone injury.

Teeth involved in bone trauma generally have a favorable prognosis, with 9 out of 10 remaining in the oral cavity after 3 years of follow‐up. Additionally, one‐third of these teeth showed no complications during the follow‐up period. PN was the most frequent outcome, affecting approximately half of the teeth evaluated at the 3‐year mark.

Alveolar bone avulsion was associated with an increased risk of inflammatory resorption, PN, and failure of the monitored teeth. Furthermore, teeth that were reimplanted or repositioned after trauma showed an elevated risk of inflammatory resorption, replacement resorption, and tooth loss, making these the variables most strongly linked to a greater number of adverse healing outcomes.

## Author Contributions

**Cristina Braga Xavier:** conceptualization, methodology, data curation, validation, formal analysis, writing – original draft, writing – review and editing, and project administration. **Anthony Marcowich Rocha:** investigation, data curation, writing – review and editing. **Kerian Dorothy Rehbien:** conceptualization, methodology, data curation, and writing. **Lucas Borin Moura:** conceptualization, methodology. **Letícia Kirst Post:** conceptualization and methodology. **Kauê Collares:** methodology, investigation, validation, formal analysis, writing – review and editing, supervision.

## Ethics Statement

Ethical approval for this research was obtained from the Research Ethics Committee of the School of Dentistry at the Federal University of Pelotas under protocol no. 2.419.514.

## Conflicts of Interest Statement

The authors declare no conflicts of interest.

## Supporting information


**Data S1:** edt70012‐sup‐0001‐TableS1.docx.

## Data Availability

The data that support the findings of this study are available from the corresponding author upon reasonable request.
