# Supplementary material for: Survival and Success of Teeth Involved in Alveolar Bone Injuries: Up to 10 Years of Follow‐Up
Source: Dent Traumatol. 2025 Aug 28;42(2):176–86. doi: 10.1111/edt.70012 (PMC12990844; doi:10.1111/edt.70012)
Supplement: Supplementary file 1 — Data S1: edt70012‐sup‐0001‐TableS1.docx. [file EDT-42-176-s001.docx]

| Supplementary table. Results of univariated Cox’s regression analysis with shared frailty for variables related to success and healing complications (N=145 teeth in 47 patients) | | | | | | | | | | | | |
| --- | --- | --- | --- | --- | --- | --- | --- | --- | --- | --- | --- | --- |
|  | Success ^a^ | | Calcificação | | Anquilose | | Reabsorção | | Necrose ^b^ | | Tooth loss ^b^ | |
|  | HR (95% CI) | p-value | HR (95% CI) | p-value | HR (95% CI) | p-value | HR (95% CI) | p-value | HR (95% CI) | p-value | HR (95% CI) | p-value |
| *Patient-related variables* |  |  |  |  |  |  |  |  |  |  |  |  |
| Sex (ref = Female) |  | 0.55 |  | 0,38 |  | 0,76 |  | 0,56 |  | 0,90 |  | 0,74 |
| Male | 0,8 (0,4-1,7) |  | 0,4 (0,1-3,1) |  | 1,4 (0,2-9,2) |  | 1,5 (0,4-5,3) |  | 1,1 (0,3-3,6) |  | 1,6 (0,1-21,5) |  |
| Age (ref = 7-12) |  | 0,45 |  | 0,17 |  | 0,49 |  | 0,12 |  | 0,05 |  | 0,73 |
| 13–18 | 1,9 (0,7-5,2) |  | 0,4 (0,1-5,9) |  | 0,7 (0,1-6,0) |  | 4,0 (0,4-39,4) |  | 10,7 (1,6-72,7) |  | 0,2 (0,1-8,7) |  |
| 18 ou mais | 1,5 (0,7-3,4) |  | 0,1 (0,1-1,1) |  | 0,3 (0,1-2,2) |  | 7,7 (0,9-64,0) |  | 6,7 (1,2-38,7) |  | 0,5 (0,1-6,7) |  |
| Etiologia (ref = Queda) |  | 0,99 |  | 0,98 |  | 0,77 |  | 0,61 |  | 0,60 |  | 0,69 |
| Automobilistico | 0.9 (0,4-2,1) |  | 1,0 (0,1-12,8) |  | 0,4 (0,1-2,2) |  | 1,4 (0,3-5,5) |  | 2,2 (0,4-11,7) |  | 0,2 (0,1-3,6) |  |
| Violencia | 1.0 (0,4-2,7) |  | 0,6 (0,1-10,8) |  | * |  | 0,6 (0,1-3,5) |  | 3,5 (0,6-21,9) |  | 0,5 (0,1-10,3) |  |
| Outros | 1.0 (0,4-2,8) |  | 1,0 (0,1-26.2) |  | 0,4 (0,1-3,3) |  | 1,8 (0,4-8,3) |  | 2,7 (0,4-16,8) |  | 0,8 (0,1-16,0) |  |
| Número de dentes envolvidos no trauma (tercil) (ref = 1 a 3) |  | 0,67 |  | 0,59 |  | 0,18 |  | 0,20 |  | 0,68 |  | 0,99 |
| 4 a 5 | 0,8 (0,4-1,6) |  | 0,6 (0,1-3,7) |  | 0,3 (0,1-1,1) |  | 0,6 (0,2-1,7) |  | 1,6 (0,5-5,2) |  | 1,1 (0,1-12,3) |  |
| 6 a 9 | 0,7 (0,3-1,7) |  | * |  | * |  | 1,7 (0,5-6,4) |  | 1,0 (0,2-5,0) |  | 1,2 (0,1-30,2) |  |
| Arco envolvido no trauma (ref = Apenas 1) |  | 0,46 |  | 1,00 |  | 0,48 |  | 0,60 |  | 0,92 |  | 0,24 |
| Ambos | 0,8 (0,4-1,5) |  | * |  | 0,5 (0,1-3,0) |  | 0,8 (0,3-2,2) |  | 0,9 (0,3-2,9) |  | 3,7 (0,4-32,5) |  |
| Tempo atendimento (ref = até 1 semana) |  | 0,83 |  | 0,46 |  | 0,33 |  | 0,74 |  | 0,82 |  | 0,80 |
| Após 1 semana | 0,9 (0,5-1,8) |  | 0,5 (0,1-3,5) |  | 2,2 (0,5-10,5) |  | 1,2 (0,4-3,2) |  | 0,9 (0,3-2,7) |  | 0,7 (0,1-7,4) |  |
| Ortodontia (ref = não) |  | 0,24 |  | 1,00 |  | 0,63 |  | 0,35 |  | 0,56 |  | 1,00 |
| Sim | 0,6 (0,2-1,4) |  | * |  | 0,5 (0,1-6,5) |  | 1,8 (0,5-5,9) |  | 0,6 (0,2-2,8) |  | * |  |
| Tipo de fratura ossea (ref = Tábua e bloco) |  | 0,008 |  | 0,81 |  | 0,89 |  | 0,03 |  | 0,06 |  | 0,17 |
| Avulsão | 2,8 (1,3-6,0) |  | 1,5 (0,1-39,4) |  | 1,2 (0,1-9,6) |  | 3,2 (1,2-8,8) |  | 3,0 (1,0-9,5) |  | 5,5 (0,5-64,9) |  |
| Número de dentes envolvidos na fratura (tercil) (ref = 1 a2) |  | 0,75 |  | 0,27 |  | 0,10 |  | 0,07 |  | 0,31 |  | 0,93 |
| 3 a 4 | 0,9 (0,4-1,7) |  | 0,3 (0,1-1,3) |  | 0,2 (0,1-0,9) |  | 0,4 (0,2-1,0) |  | 2,5 (0,7-8,1) |  | 0,8 (0,1-8,6) |  |
| 5 a 7 | 0,7 (0,2-2,0) |  | * |  | * |  | 0,2 (0,2-1,4) |  | 2,7 (0,4-19,9) |  | 1,5 (0,1-58,4) |  |
| Perda dentária inicial (ref = não) |  | 0,43 |  | 0,95 |  | 0,58 |  | 0,88 |  | 0,16 |  | 0,58 |
| Sim | 1,4 (0,6-3,5) |  | 0,9 (0,1-19,9) |  | 0,5 (0,1-5,6) |  | 1,1 (0,3-3,7) |  | 2,5 (0,7-9,1) |  | 2,1 (0,1-29,9) |  |
| *Tooth-related variables* |  |  |  |  |  |  |  |  |  |  |  |  |
| Tooth (ref = Lateral/canine) |  | 0.001 |  | 0,30 |  | 0,02 |  | 0,19 | xxx | 0,008 |  | 0,33 |
| Central | 2.2 (1.3-3.6) |  | 1,9 (0,6-6,8) |  | 6,3 (1,3-31,3) |  | 1,7 (0,8-3,8) |  | 2,6 (1,3-5,4) |  | 1,8 (0,5-6,1) |  |
| Dental arch (ref = lower) |  | 0.88 |  | 0,24 |  | 0,03 |  | 0,21 |  | 0,40 |  | 0,81 |
| Upper | 1,1 (0,6-2,0) |  | 3,2 (0,5-21,0) |  | 10,6 (1,2-90,4) |  | 1,8 (0,7-4,8) |  | 0,6 (0,2-1,8) |  | 1,3 (0,1-11,5) |  |
| Trauma dentário (ref = Dentário) |  | 0,28 |  | 0,033 |  | >0,001 |  | 0,29 |  | 0,93 |  | 0,40 |
| Sustentação | 1,5 (0,8-2,8) |  | 30,5 (2,3-402,1) |  | 1,43 (0,1-17,4) |  | 1,8 (0,6-5,3) |  | 1,2 (0,5-3,0) |  | 1,5 (0,2-10,9) |  |
| Avulsão | 2,0 (0,8-4,9) |  | 4,1 (0,2-100,6) |  | 52,9 (5,1-545,4) |  | 3,3 (0,7-14,7) |  | * |  | 6,0 (0,4-84,7) |  |
| Fratura radicular (ref = não) |  | 0,24 |  | 0,94 |  | 1,00 |  | 0,19 |  | 0,31 |  | 0,84 |
| Sim | 1,6 (0,7-3,5) |  | 0,9 (0,1-10,2) |  | * |  | 2,2 (0,7-6,9) |  | 1,8 (0,6-5,8) |  | 1,2 (0,2-6,0) |  |
| Envolv. LP (ref = não) |  | 0.26 |  | 0,89 |  | 0,94 |  | 0,73 |  | 0,18 |  | 0,52 |
| Sim | 1,5 (0,8-2,9) |  | 1,2 (0,2-8,6) |  | 0,9 (0,2-4,8) |  | 1,2 (0,4-3,3) |  | 2,1 (0,7-6,3) |  | 0,5 (0,1-3,5) |  |
| Rizogenese (ref =Completa) |  | 0,84 |  | 0,71 |  | 0,32 |  | 0,95 |  | 0,71 |  | 1,00 |
| Incompleta | 0,8 (0,2-3,0) |  | 0,6 (0,1-7,9) |  | 4,3 (0,2-80,3) |  | 1,1 (0,1-11,7) |  | 0,6 (0,1-7,7) |  | * |  |
| Uso de Contenção (ref = não) |  | 0,32 |  | 0,89 |  | 0,54 |  | 0,93 |  | 0,27 |  | 0,77 |
| Sim, até 60 dias | 0,7 (0,3-1,7) |  | * |  | 2,5 (0,3-22,7) |  | 0,8 (0,2-2,9) |  | 0,9 (0,2-3,3) |  | 2,7 (0,2-47,5) |  |
| Sim, mais de 60 dias | 1,3 (0,6-2,8) |  | 0,6 (0,1-4,1) |  | 3,1 (0,4-22,3) |  | 0,9 (0,3-2,9) |  | 2,2 (0,7-7,2) |  | 2,0 (0,2-23,8) |  |
| Reimplante/ Reposicionamento (ref = não) |  | 0,29 |  | 0,97 |  | >0,001 |  | 0,02 |  | ---- |  | 0,02 |
| Sim | 1,4 (0,7-2,8) |  | 1,1 (0,1-13,2) |  | 23,0 (4,6-114,8) |  | 3,6 (1,3-10,3) |  | * |  | 15,2 (1,5-150,1) |  |
| HR: Hazard ratio; CI: Confidence interval; ref = reference.  ^a^ Tooth that did not show any outcome in period;  ^b^ For the outcome of pulp necrosis, 114 teeth were evaluated, considering that 31 teeth underwent endodontic treatment at the beginning of the follow-up.  ^*^ The outcome does not have enough failures to perform a multivariate analysis. | | | | | | | | | | | | |
